# Managing antimicrobial resistance in a changing climate: Insights from a workshop

**DOI:** 10.4102/jphia.v17i1.1486

**Published:** 2026-01-12

**Authors:** Vrinda Nampoothiri, Candice Bonaconsa, Deepshikha Batheja, Sipho Dlamini, Anastasia Koch, Esmita Charani

**Affiliations:** 1 Amrita Institute of Medical Sciences, Amrita Vishwa Vidyapeetham, Faridabad, Haryana, India; 2 Division of Infectious Diseases and HIV Medicine, Department of Medicine, University of Cape Town, Cape Town, South Africa; 3 Indian School of Business, Hyderabad, India; 4 One Health Trust, Bengaluru, India; 5 Isivivana Centre, Khayelitsha, Cape Town, South Africa; 6 Molecular Mycobacteriology Research Unit, Department of Pathology, Faculty of Health Sciences, University of Cape Town, Cape Town, South Africa; 7 Institute of Infectious Disease and Molecular Medicine, Faculty of Health Sciences, University of Cape Town, Cape Town, South Africa

## Introduction

Bacterial antimicrobial resistance (AMR) has been identified as a significant public health and developmental threat worldwide. The recent Global Research on AMR (GRAM) project reported that AMR has claimed at least 1 million deaths each year since 1990, with more than 21% of the global total reported from Africa.^1^ The second United Nations General Assembly (UNGA) high-level meeting on AMR committed to: (1) reducing the number of global deaths from bacterial AMR by 10% by 2030; (2) implementation of National Action Plans (NAPs) through a One Health approach; and (3) establishment of an independent panel for evidence-based action against AMR. A central premise was the recognition that high-quality, relevant data are essential for supporting researchers and policymakers and for enhancing awareness and communication around AMR.^2^ Notably, Article 14 of the UNGA declaration highlights the need to consider the intersection of AMR with gender equity and climate change, particularly in vulnerable populations. Furthermore, Article 46 advocates a multisectoral approach to mitigate AMR.^2^

The AMR burden is higher in low- and middle-income countries (LMICs), where resistance patterns vary significantly.^1^ It is important to use an intersectional lens and identify how vulnerability shaped by socio-cultural and structural conditions of inequity, such as gender, race and caste, drives the exposure to and spread of AMR.^3^ Women and girls have limitations accessing care in many societies, making them prone to developing drug-resistant infections. Additionally, gendered roles in caregiving, food preparation and water collection increase the risk of exposure to drug-resistant infections.^4^ Increasing evidence is being generated on the intersection of gender and AMR, which highlights that persistent gender discrimination exacerbates the key drivers of AMR, including misuse of antibiotics, insufficient water, hygiene and sanitation (WASH), as well as infection control practices.^5^ The World Health Organization (WHO) report on gender and AMR highlights how gender influences access to health care and the emergence, spread and response to drug-resistant infections.^6^

Climate change is likely to have major implications on the effects of AMR. Extreme weather events disrupt sanitation systems, leading to outbreaks of waterborne diseases that will need antibiotic treatment^7^ that could destabilise access to overburdened healthcare services. Moreover, a recent modelling study has shown that responding to socioeconomic factors will be most effective to address the dual challenges of climate change and AMR.^8^ These studies demonstrate emerging evidence for how climate change and social determinants of health will interact to exacerbate AMR.

Inequitable access to antibiotics, challenges in disseminating clear and actionable health information and limited mechanisms for engaging communities in decision-making processes can undermine the success of interventions such as improved WASH, vaccination programmes and infection prevention.^3,9^ The impact of these interventions varies across social stratifiers, including gender.^10^ The response to these complex and multifaceted issues requires an integrated One Health approach as well as close collaboration across sectors and regions.

In 2023, the WHO Training in Tropical Diseases (TDR), the Special Programme for Research and Training in Tropical Diseases, launched a call for proposals for ‘Gender, antimicrobial resistance and climate change threat to human health in the context of infectious diseases of poverty’. The aim was to identify factors affecting health interventions amid climate change and AMR. As grant recipients, we held intersectoral and interdisciplinary workshops to explore the intersection of AMR, gender and climate through an equity lens: three in India and one in South Africa. This *Public Health Picture* describes the process and outcomes of the final workshop, held in Cape Town, South Africa on 19 July 2024, where experts shared real-world insights that underscored the need for integrated, cross-sectoral approaches.

## Participant selection and workshop outline

To promote open dialogue and identify key problems and solutions, we brought together a range of perspectives. This workshop, held in South Africa, included participants from the region, purposively selected through established professional networks to ensure disciplinary diversity (Table 1). Workshop participant expertise included infectious disease, health leadership, climate change, gender research and public health. Contributors from India, involved in earlier workshops, joined to provide continuity and support. One attendee, based in the United Kingdom, works across South Africa and India. Although we aimed to reflect a range of diverse social identities, particularly across race, ethnicity and gender, a limitation in our reporting is that we did not ask attendees to self-identify these characteristics, which restricts our ability to confirm the representativeness of the group.

**TABLE 1:** Details of the workshop participants (*N* = 49).

Variables	South Africa		India		United Kingdom	
	*n*	%	*n*	%	*n*	%
Number of participants per country	45	92	3	6	1	2
**Professional backgrounds**						
Clinical and public health (*n* = 29)	28	97	1	3	0	-
Community and public engagement (*n* = 10)	10	100	0	-	0	-
Social science (*n* = 5)	2	40	2	40	1	20
Climate systems and data analysis (*n* = 5)	5	100	0	-	0	-

**FIGURE 1:**
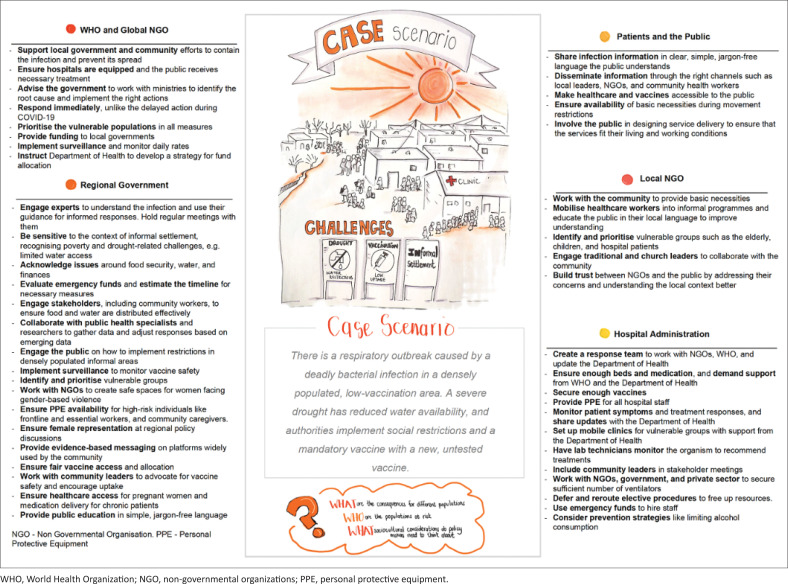
Recommendations provided by various stakeholder groups in response to the case scenario activity.

Seven presentations from different sectors and countries covered the key intersectional drivers of AMR, gender and climate change. A hypothetical case-based reflective session facilitated cross-disciplinary exchange of expert insights. Participants were randomly divided into five stakeholder groups: WHO and global non-governmental organisation (NGO), regional government, local NGO, hospital administration and patients and/or the public. Each group explored the case scenario from their stakeholder’s perspective, guided by specific questions (Figure 1) and applied an intersectional lens to their discussion. Asking people to contribute from perspectives different from their own stimulated thinking beyond traditional expertise and encouraged the consideration of intersectionality. A facilitated discussion across the stakeholder groups followed. Analysis of the workshop recordings revealed several themes that are described below and illustrated with participant quotes in Table 2.

**TABLE 2:** Table of quotes from the speaker input and group discussions at the interdisciplinary workshop on the intersection of antimicrobial resistance, gender and climate.

Theme	Quote number	Quote
The role of governance in health policy	Q1	‘It’s often challenging due to limited resources and the small amount of people driving change. Why are researchers the ones bringing this to the table? We should consider how things are done, and how to better support policymakers with the resources needed to drive the change.’ (Community and public engagement expert, Participant 42, Female)
	Q2	‘Governments ask academic institutions how to engage with communities and draft policies so why do we then have health clinic committees? I run a mentorship programme in the district, training health committee members on policies like the National Strategic Plan on Tuberculosis (TB), Human Immunodeficiency Virus (HIV) and Sexually Transmitted Infections (STIs), as well as the TB recovery plan because the government requires these committees.’ (Clinical and public health expert, Participant 30, Male)
The role of social determinants of health and AMR	Q3	‘The marginalised groups also have less access to toilet facilities. Open defecation is still widely practised [*India*], and we can see only 68% of the people from Scheduled Tribe (ST) households have access to toilets, compared to 92% of upper caste households.’ (Social science expert, Participant 5, Female)
	Q4	‘Households from Scheduled Caste (SC) and ST groups often lack access to safe and hygienic menstrual products. Women from ST households also have among the highest prevalence of STIs while demonstrating the least awareness of them. Regarding pregnancy, women from SC/ST and Other Backward Caste (OBC) groups have lower access to quality reproductive health services, and those from lower castes tend to become mothers at younger ages than those from higher castes.’ (Social science expert, Participant 5, Female)
Access to antibiotics	Q5	‘There are laws against giving antibiotics, but pharmacists say that the public insist, and so they comply. Patients told us that for mild illnesses, they are going to the pharmacies instead of doctors due to the cost and days lost, especially, and then the doctors may not give antibiotics.’ (Social science expert, Participant 5, Female)
	Q6	‘Could it be that people misunderstand symptoms and seek antimicrobials for the wrong reasons? This highlights an educational gap and raises questions about responsibility. For me, it’s less about interrogating policy and more about engagement and getting those taking, prescribing, and distributing antimicrobials to fully understand their use. We can then slowly move to accountability by patients, prescribers, farmers and accountability by the government.’ (Clinical and public health expert, Participant 28, Male)
Language barriers for inclusive and effective healthcare communication in AMR	Q7	‘The use of antibiotics and language is especially important in some of our diverse populations. A key issue is language barriers. Someone may ask for something to “clean their blood” but the translation could lead to them receiving something entirely different.’ (Clinical and public health expert, Participant 33, Female)
	Q8	‘From an anthropological perspective, patients associated resistance with their own bodies being resistant to the antibiotic, rather than the bacteria. This idea comes from HIV and AIDS. Overcoming this misconception, irrespective of gender, will be a complicated challenge if we do not get it right.’ (Clinical and public health expert, Participant 33, Female)
	Q9	‘And if we think about the world of TB, TB partnerships [*have*] published guidelines on appropriate language-avoiding terms like “default” or “loss to follow-up.” Similarly, we need to consider whether we are referring to antibiotics or is it antimicrobial[*s*]? X [*the name of an ID physician was mentioned*] has published on this, and I believe it’s an important discussion to have.’ (Clinical and public health expert, Participant 30, Male)
	Q10	‘The language we used was inclusive and accessible. It would be valuable to involve more science communicators, and others already working in this space. Many resources exist, for example the TB framework on appropriate language. A lot of what we’re talking about exists in one form or another, but it’s not accessible.’ (Community and public engagement expert, Participant 47, Female)
Social listening and community engagement	Q11	‘For me, what has come across all the discussions has been inclusivity and communication. First, we must make sure that we equip ourselves with data. And second, we must involve the communities we aim to support and ensure [*that*] our communication reflects their experiences and what they believe should happen for their own communities. This is what struck me the most.’ (Clinical and public health expert, Participant 28, Male)
	Q12	‘The problem is that policies are failing because there’s no deliberate community and public engagement around things that matter. And if we believe this matters, then the qualitative aspects are absolutely critical.’ (Clinical and public health expert, Participant 28, Male)
	Q13	‘During my Masters studies, I conducted a study asking communities about major diseases, yet none mentioned diarrhoea, even though they were spending 20–25% of their monthly income on treatment [*for it*]. When I translated this into cost savings and demonstrated the financial benefit and highlighted the need to go to the hospital rather than buying from the roadside, they saw how much money they were saving. Then, the hospital became “their friend.” I don’t know if this shift continued, but as long as people recognise the financial impact of their choices, sustainability is possible.’ (Clinical and public health expert, Participant 46, Male)
	Q14	‘I think there’s still a lot of work to be done around AMR as a topic. Most of my engagement has been around TB, but I don’t think people fully recognise when they are impacted by AMR. Public education is important, but listening to people is what will solve their challenges around this topic. Perhaps the priority is not AMR itself but improving access to care before shifting policy. There is also an important link to climate and the environment.’ (Community and public engagement expert, Participant 3, Female)
Intersectoral collaboration	Q15	‘There is strong buy-in from communities, but what is lacking is engagement from the decision-makers. It’s time to bring them into the discussions, because while sharing data with the community and everyone is valuable, many of the necessary changes must come from government.’ (Community and public engagement expert, Participant 17, Female)
	Q16	‘For me, the issue remains the huge disconnect between policy, clinical practice and the community. The real challenge is the “us and them” mentality. It’s not just about poor communities, if policies are bad, they affect everyone, regardless of wealth. In [South Africa], where we are invested in this kind of care treatment, we need to see ourselves as part of the community, as people impacted by these policies, and as those responsible for education, and at all levels and across all sectors.’ (Clinical and public health expert, Participant 14, Female)
	Q17	‘Our health system needs better information on a lot of things. This applies to both our new generation of doctors and the more experienced practitioners. Often, doctors rely on their experience, saying: “I’ve been doing this thing and its works,” making it difficult to enforce adherence to policies.’ (Clinical and public health expert, Participant 46, Male)

AMR, antimicrobial resistance; AIDS, acquired immunodeficiency syndrome.

## Key considerations about antimicrobial resistance, gender, and climate

### The need for intersectional research in antimicrobial resistance

The intersection of gender and climate change was reiterated and highlighted how social norms and socio-cultural factors influence access to health interventions. The importance of inclusive policies that consider gender inequality and power dynamics was underscored:

‘Socio-cultural norms within countries and cultures, and how that influences people’s access to interventions are often influenced by intersectional factors such as gender, race, ethnicity, and the amount of power that gives individuals within society, within the household, within the workforce, and how that how power manifests.’ (Clinical and public health expert, Participant 1, Female)

### Impact of environment and climate change on health

Climate change impacts people’s health (morbidity and mortality), income, mental health, education and livelihood, including basic needs such as access to food. The impact of climate change is found to be more pronounced in marginalised or vulnerable communities, like women and children:

‘Whilst climate change affects everyone, most affected are women because they’re primary caregivers in our homes. As much as people say we’re all affected by climate change, women and children are the most affected by climate change.’ (Community and public engagement expert, Participant 13, Female)

Despite emerging awareness of the issues around climate change, there is not much action being taken to reduce their occurrence in the future. Underserved communities face heightened challenges because of poor sanitation, undignified housing and proximity to livestock in rural areas. While there is a lack of community representation and voices in the discourse around climate change, projects do exist that empower communities through science communication and co-development of solutions, addressing health and environmental sustainability.

### The role of governance in health policy

Discussions highlighted the limited change occurring in the communities where AMR burden is greatest and most vulnerable to climate change. For change to happen, it is important that we include policy decision-makers in discussions around key issues that impact communities:

‘It’s time for us to start including these decision-makers in our talks, because as much as we can, you can share the data with the community and everyone, but there are changes happening that should be done by government.’ (Community and public engagement expert, Participant 17, Female)

Limited human and other resources hinder effective policy change. Additionally, even when policymakers are interested and committed to engaging with the people affected by health challenges, they do not have the skills or tools to facilitate these engagements. It is therefore important to also consider how policymakers can be supported to bring about these changes (Q1, Table 2). Policymakers often depend on academic institutions to advise on and implement how community engagement should be done (Q2, Table 2).

### The role of social determinants of health and antimicrobial resistance

Key social determinants impacting AMR were recognised to be gender, race and income. To deepen understanding of systemic inequities experienced in a different context, a workshop facilitator from India presented on caste-based disparities. Historically, the four caste systems, structured around occupational hierarchies, were reclassified under British rule into general categories, scheduled castes, scheduled tribes and other backward castes.^9^ Other than the general category, members of the other categories are employed in manual scavenging, sewage cleaning, fishing and domestic cleaning, all of which increase their risk of infections. Members of these castes also have restricted access to healthcare, health information and good nutrition:

‘Caste hierarchies also limit healthcare access and limit the spread of health information. In each of the determinants, lower caste groups have high disease, because of their [*poor*] nutrition. There are underlying determinants which relate to WASH, vaccines, health promotion schemes and discrimination.’ (Social science expert, Participant 5, Female)

People in these circumstances also have limited access to toilets and continue to practise open defecation, further increasing the risk of infection (Q3, Table 2). The risk is higher in women, as they are the ones who perform domestic chores, which exposes them to contact with contaminated water, food handling, preparation of uncooked meat, fish and fresh produce and using traditional cooking methods. Women from lower caste groups also have a lack of access to proper menstrual hygiene and reproductive health care and are at an increased risk of sexually transmitted diseases, as they are more impacted by sexual violence (Q4, Table 2).

### Access to antibiotics

Participants highlighted that the underlying approaches towards AMR mitigation, including antibiotic stewardship programmes, have been utilitarian at times, with the assumption that everyone has equal access to universal healthcare, including antibiotics. While some countries have greater access to innovations, diagnostic tests, vaccines and antibiotics, many countries facing the greatest burden of AMR do not have the same or any access at all. Globally, although not as much of an issue in South Africa, it was recognised that over-the-counter (OTC) dispensing of antibiotics is a major contributing factor to AMR. In many parts of the world, buying an antibiotic from a pharmacy is the more accessible means to healthcare compared to visiting a general practitioner:

‘It’s the same dilemma in [*India*] about access to antibiotics. So, it’s much cheaper to buy an antibiotic than it is to go and see a doctor or to do a diagnostic test to find out whether your condition is bacterial to begin with.’ (Clinical and public health expert, Participant 33, Female)

Often, when individuals do have access to a doctor and an antibiotic is not prescribed, it can cause further delays in curing the illness and days of earnings lost because of absence from work (Q5, Table 2). The public needs to be better engaged in why the safe use of antibiotics is important. Participants highlighted that before thinking about strategies to manage AMR, it is important that the people experiencing the consequences of infections are listened to (Q6, Table 2).

### Barriers for inclusive and effective healthcare communication in antimicrobial resistance

Communication barriers can lead to miscommunication between healthcare workers, patients and the public, resulting in the dispensing of different treatments than requested by the patient (Q7, Table 2). Many people, regardless of the language they speak, do not understand the term ‘antibiotic resistance’:

‘Terminology used for antibiotics, for resistance, for infection control, and we went through all the official languages, there’s a big problem here from a language point of view and even in English, people don’t understand it.’ (Clinical and public health expert, Participant 12, Male)

There are varied beliefs about what these medicalised terms mean, with misconceptions that peoples’ bodies become resistant to antibiotics rather than bacteria becoming resistant (Q8, Table 2), emphasising the importance of using appropriate and precise terminology, such as distinguishing between antibiotics and antimicrobials, when communicating about AMR to the public. Recently published tuberculosis (TB) guidelines advocating for appropriate language were identified as an example of positive communication (Q9, Table 2). Improving accessibility and inclusivity through effective engagement and science communication remains an important area that requires further work (Q1, Table 2).

### Social listening and community engagement and learning

Participants’ personal reflections helped develop an understanding of the challenges faced by communities. Severe health issues associated with TB and human immunodeficiency virus (HIV), for example, which are adversely affected by inadequate sanitation and living conditions, were highlighted. The shared experiences alluded to the disproportionate burden on women and stressed the importance of involving communities in finding appropriate solutions, advocating for an approach that shifts power to the people who are directly affected.

Inclusive communication, crucial for effective healthcare access, involves active listening to and incorporating community feedback on health experience and access to build trust and ensure contextually and culturally appropriate interventions and epistemic humility (the ability and willingness to learn and unlearn by experts and non-experts alike) (Q11, Q12, Table 2). Antimicrobial resistance may not be a key issue for people who are facing a myriad of pressing social challenges, and understanding this for communication is important (Q13, Table 2). Intentional community engagement, where communities are active participants and stakeholders, is vital for developing sustainable solutions (Q14, Table 2). Emphasising the financial and health consequences of choices can encourage appropriate health care behaviours. Public education should focus on relatable issues to address the root cause of AMR, as many people do not understand the term, and in South Africa, the public may identify with more widely recognised diseases such as TB, including drug-resistant TB (DR-TB):

‘We started doing some of that work in relation to AMR. And what our very early data is telling us is that people don’t know what the word antimicrobial resistance means. People know what DR-TB means because people have experienced it. But if you go into the community and you start talking about AMR, people will just look at you.’ (Community engagement expert, Participant 3, Female)

### Intersectoral collaboration

Bringing together multi-sectoral groups to respond to healthcare issues such as AMR and infection control is vital; however, these meetings often rely on funding from research grants and research activities, making them unsustainable:

‘I think getting this multi-sectoral group of people together is refreshing. And it’s sad that it takes a grant and research to do this. And I think so much has been mentioned here today that I really think we need to move towards trying to do this more regularly as part of our day job.’ (Clinical and public health expert, Participant 38, Female)

Examples of the impact of successful intersectoral collaborations include strengthening health committees through mentorship to equip community members with knowledge to engage in health system improvements (Q2, Table 2). Multidisciplinary approaches to evidence synthesis and collaborations with NGOs that focus on sustained community discourse and engagement can inform better health policy and outcomes. Food security in socioeconomically deprived populations in many countries, as a basic need that cuts through all health challenges, was linked to climate change. While there is substantial community involvement in addressing AMR, there is a lack of engagement from decision-makers to make changes (Q15, Table 2). Participants further described the danger of an ‘us and them’ approach, thereby distancing policymakers from the community (Q16, Table 2). This highlights a disconnect between health care policy, clinical practice and communities affected by policies. Cross-sectoral work to improve engagement, accountability and policy implementation on AMR is needed to plug the gap between policy and practice around behaviours and adherence, as well as prescribing protocols (Q17, Table 2).

## Recommendations towards an intersectional view on antimicrobial resistance, gender, and climate

The case scenario and the resulting discussions are summarised in Figure 1. This exercise highlighted the interdependence of the sectors and underscored the importance of each individual sector and the need for collaboration towards a concerted effort to manage AMR as it intersects with climate change.

**FIGURE 2:**
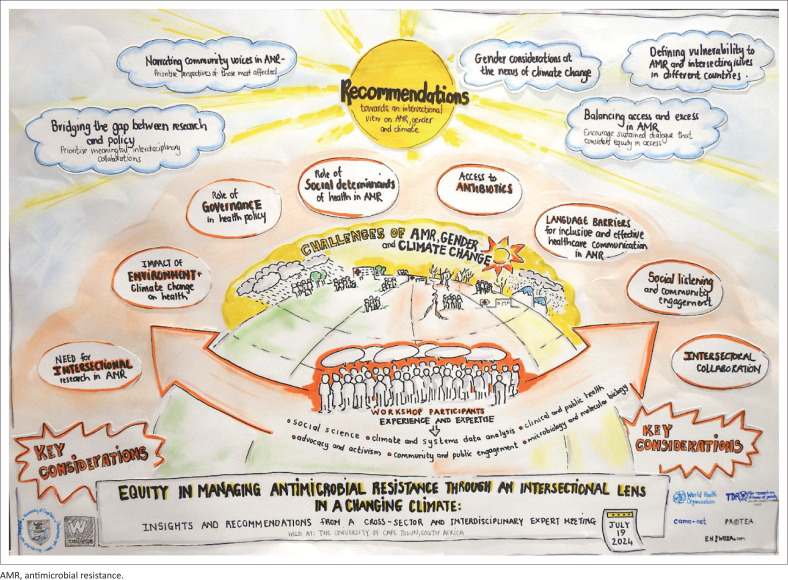
Insights and recommendations from a cross-sector and interdisciplinary expert meeting on how social determinants of health intersects with climate change and antimicrobial resistance.

From this meeting, a set of key recommendations emerged, recognising the complexity of viewing AMR through an intersectional lens. Figure 2 provides a summary of the insights and recommendations from the workshop.

### Meaningfully connecting research to policy

Meaningfully connecting research to policy requires collective action. Participants stressed the importance of interdisciplinary collaboration among researchers, healthcare professionals, policymakers and community members. Strengthening collaboration between the government, NGOs, hospitals (both public and private) and global organisations such as WHO was emphasised, potentially facilitated through grants and project funding that require multi-sectoral partnerships. Before developing policies, researchers and policymakers should undertake ‘listening exercises’ to understand the experiences of affected communities and identify potential community-led solutions, with special focus on the vulnerable in the community. Listening can be facilitated through workshops and focus groups between different sectors as well as training of lay community members to understand key research issues. Policymakers may need support and possibly even bespoke training to facilitate effective social listening and incorporation into policy.

### Community voices in antimicrobial resistance

Experiences of health and disease occur outside of the formal healthcare setting. Language used for communicating such issues with the public should be clear and accessible. The perspectives and voices of people living in communities most at risk from AMR and climate change must be central to the efforts taken to manage their health needs, rather than treated as an afterthought or a ‘tick list exercise’ to satisfy funders. For community engagement to be effective, it should be anchored in building trust, which takes time. Therefore, long-term engagement with communities that prioritise trust-building must be supported. Public education and community engagement approaches should consider the context in which people live, with the awareness that in many ‘at-risk areas’, people face a myriad of social challenges. Approaches to engaging with communities should focus on relatable issues that can encourage responses to the root causes of AMR and adaptation to climate change. Finally, lived experience should be valued as equal to academic or policy expertise within the knowledge economy.

### Gender considerations at the nexus of antimicrobial resistance and climate change

The gendered influence of climate change on AMR reflects how climate-driven vulnerabilities are unevenly distributed. Extreme weather events and water and food insecurity can intensify exposure to unsanitary conditions, increasing risks of infections that necessitate antibiotic treatment. Moreover, restricted healthcare access for women – often because of socioeconomic, cultural or systemic barriers – limits timely intervention, compounding the risks of AMR. Recognising and incorporating gender dynamics, through responsive research, data gathering and policies, in climate and health interventions is essential to reducing the broader impact of AMR in climate-affected populations.

### Balancing access and excess in antimicrobial resistance: Encouraging sustained dialogue that considers equity in access

Balancing access and excess in managing AMR is essential for health equity. In high-income settings, excessive access to antibiotics has fuelled overuse and resistance, whereas many LMICs struggle with limited or delayed access, which is likely to be exacerbated with climate shocks. This disparity highlights the need for a global dialogue on AMR that emphasises equity, ensuring that strategies for antibiotic stewardship do not inadvertently limit essential access for underserved populations. For lasting impact, decision-makers should ensure balanced distribution and stewardship practices that prioritise patient needs and local healthcare realities, reducing both overuse in high-access areas and under-treatment where resources are scarce.

### Defining vulnerability to antimicrobial resistance and intersecting issues in different countries

In LMICs, limited healthcare access and socioeconomic conditions contribute significantly to AMR risk, especially among communities with restricted access to WASH. Climate change also disproportionately impacts vulnerable populations, exacerbating AMR risks by increasing exposure to infectious agents through changing ecosystems, extreme weather and compromised WASH infrastructure. Gender dynamics add another layer of vulnerability; women in caregiving roles may face higher exposure to pathogens while simultaneously experiencing less access to healthcare resources. Effective AMR mitigation must ensure contextually tailored approaches to these intersecting vulnerabilities through equitable healthcare policies, context-specific research and targeted resource allocation that prioritises marginalised populations.

## Conclusion

Addressing AMR through an intersectional lens requires uncovering, acknowledging and acting upon prevailing vulnerabilities that exist across different communities. This cross-disciplinary meeting generated valuable real-world insights on the intersection of climate, gender equity and AMR, emphasising the importance of interconnectedness and the value of collaborating across sectoral and disciplinary silos. The recommendations provide contextual knowledge that delivers to the United Nations declaration on AMR, particularly for populations most at risk.
